# Accuracy of an artificial intelligence-enabled diagnostic assistance device in recognizing normal chest radiographs: a service evaluation

**DOI:** 10.1093/bjro/tzae029

**Published:** 2024-09-14

**Authors:** Amrita Kumar, Puja Patel, Dennis Robert, Shamie Kumar, Aneesh Khetani, Bhargava Reddy, Anumeha Srivastava

**Affiliations:** Frimley Health NHS Foundation Trust, Frimley, United Kingdom; Frimley Health NHS Foundation Trust, Frimley, United Kingdom; Qure.ai, Bangalore, India; Qure.ai, London, United Kingdom; Qure.ai, London, United Kingdom; Qure.ai, Mumbai, India; Qure.ai, Mumbai, India

**Keywords:** artificial intelligence, radiology, chest X-ray, computer-assisted diagnosis, autonormal chest X-ray reporting

## Abstract

**Objectives:**

Artificial intelligence (AI) enabled devices may be able to optimize radiologists’ productivity by identifying normal and abnormal chest X-rays (CXRs) for triaging. In this service evaluation, we investigated the accuracy of one such AI device (qXR).

**Methods:**

A randomly sampled subset of general practice and outpatient-referred frontal CXRs from a National Health Service Trust was collected retrospectively from examinations conducted during November 2022 to January 2023. Ground truth was established by consensus between 2 radiologists. The main objective was to estimate negative predictive value (NPV) of AI.

**Results:**

A total of 522 CXRs (458 [87.74%] normal CXRs) from 522 patients (median age, 64 years [IQR, 49-77]; 305 [58.43%] female) were analysed. AI predicted 348 CXRs as normal, of which 346 were truly normal (NPV: 99.43% [95% CI, 97.94-99.93]). The sensitivity, specificity, positive predictive value, and area under the ROC curve of AI were found to be 96.88% (95% CI, 89.16-99.62), 75.55% (95% CI, 71.34-79.42), 35.63% (95% CI, 28.53-43.23), and 91.92% (95% CI, 89.38-94.45), respectively. A sensitivity analysis was conducted to estimate NPV by varying assumptions of the prevalence of normal CXRs. The NPV ranged from 88.96% to 99.54% as prevalence increased.

**Conclusions:**

The AI device recognized normal CXRs with high NPV and has the potential to increase radiologists’ productivity.

**Advances in knowledge:**

There is a need for more evidence on the utility of AI-enabled devices in identifying normal CXRs. This work adds to such limited evidence and enables researchers to plan studies to further evaluate the impact of such devices.

## Introduction

Chest radiography (chest X-ray or CXR) is the most common radiological investigation done on patients. It is an important diagnostic tool to examine airways, lung parenchyma, mediastinum, pleura, chest wall, and heart.^1^^,^^2^ According to the Statistical Report of Diagnostic Imaging Dataset published by the National Health Service (NHS) England, CXR images constitute the most commonly requested test by general practitioners (GPs), and in January 2023 alone, approximately 197000 CXR examinations were requested by GPs alone and about 663000 from all referrals.^3^ The percentage of CXR examinations reported on the same day of the test ranged only from 22% to 26% in the NHS.^3^ Many CXR examinations remain unreported for weeks due to increased demand and reduced reporting capacity.^4^ Further, this can delay optimal patient management and time to treatment. The increase in the workload of radiologists due to the high volume of radiological investigations, including CXR examinations, can result in burnout and, as a consequence, a reduction in their diagnostic accuracy.^5–7^

Artificial intelligence (AI)-enabled computer-assisted detection (CAD) software devices have the potential to identify multiple abnormalities in CXR images and separate normal from abnormal CXR images. Multiple such AI algorithms have been developed to detect abnormal radiological findings in CXR images.^8–13^ AI can function as an assistant to radiologists or as an independent reader in triaging normal CXR images from radiologists’ worklists. This can help in improving workflow efficiency, reducing the risk of burnout, and thereby allowing radiologists to spend more time on complex tasks such as interpreting more complex imaging studies.^9^^,^^14–18^ For routine clinical use, these devices need to be approved by corresponding regulatory bodies, and the number of such regulatory-cleared AI algorithms is relatively much smaller as compared to those in the “pre-approval” phase. It is important to evaluate the real-world performance of these algorithms, even if they are regulatory-cleared, to generate more evidence on the usefulness of such solutions. Recently, the National Institute for Health and Care Excellence (NICE) published an early value assessment of using AI in analysing CXRs and recommended generating more evidence on the diagnostic accuracy of AI to identify normal CXRs.^19^

We evaluated the performance of one such regulatory-cleared AI software device, named qXR, which is approved for the detection of multiple radiological findings in frontal CXR images, including classifying them as normal or abnormal.^12^^,^^20^ Triaging normal CXR images is one of the intended uses of the qXR as per CE (European Conformity) certification. This service evaluation was conducted in an NHS Trust in the United Kingdom (UK). It was focused on investigating the accuracy of qXR in the triaging of normal CXR images using CXR images from GP and outpatient (OP) referral sources from multiple hospitals under the Trust.^21^

## Methods

### About the AI software device

qXR version 3.2 (developed by Qure.ai) is a CE marked medical device. It consists of multiple deep-learning classifier models for the detection of several radiological abnormalities, which, in effect, works like a multi-label classifier as a whole. It is trained using a set of approximately 4 million chest radiograph images labelled by radiologists. All the classifier models in qXR are developed using U-Net convolutional neural networks (CNN) architecture with an EfficientNetV2 backbone.^12^ The training data for qXR contained both inpatient and OP CXRs from India (∼66%), Europe (∼20%), and United States (∼11%), and the rest of the CXRs were sourced from multiple geographies including Brazil and Vietnam. Roughly about 48% of the CXRs in the training data were from patients aged <45 years and about 60% of the total CXRs were from male patients. A diagnostic accuracy evaluation of an older version of the device based on a ground truth generated from a panel of radiologists has been reported earlier.^20^ The device can detect multiple radiological abnormalities present in lung parenchyma, pleural cavity, mediastinum, heart, and ribs in posterior-anterior and anterior-posterior (PA/AP) CXR images. In addition, the device can also classify a CXR as normal or abnormal and outputs a probability score between 0 and 1, indicating the probability of a CXR being abnormal.

### Data collection

The device was initially deployed as part of a pilot engagement in an NHS Trust. During this pilot engagement, the AI software was used in a “shadow mode” without affecting the standard-of-care. The intention of this pilot engagement was to get clinical feedback from the radiologists on a case-to-case basis. The service evaluation of the device was planned to be conducted retrospectively by collecting a randomly sampled subset of CXR images from a larger pool of consecutive CXR examinations done during the “shadow mode” period November 2022 to January 2023 from GP and OP sources. Inclusion criteria were CXR images from patients aged more than or equal to 18 years, PA/AP view, source of CXR being GP/OP, and availability of ground truth for facilitating statistical analysis. Lateral view CXR images, CXR images of patients <18 years old, duplicate CXR images, and those with a lack of ground truth were excluded. The CXR images were sourced from 6 hospitals under the Trust. These CXRs were originally acquired using multiple CXR hardware manufacturers. The AI software device then processed all CXR images, and the probability scores from the device for normal versus abnormal classification for each CXR image were obtained for further analysis.

### Ground truth

Two radiologists, a breast radiologist with 10 years of experience and a general radiologist with interest in chest imaging with 1 year of experience, reviewed all selected CXR images independently and classified each CXR as normal or abnormal. If any remarkable radiological abnormalities were detected in the CXR image, then that CXR was classified as “abnormal.” Normal CXRs were categorized as those CXRs that required no further imaging management or follow-up. Any discordance was resolved by a consensus meeting between them. During this process, they also reviewed the original/historical radiology report of the CXR but were blinded to the AI output during the process of ground truth generation. The final radiologist consensus of whether each selected CXR was normal or abnormal was used as the ground truth for all statistical analyses.

### Statistical analysis

The objective of this service evaluation was to investigate the accuracy of the AI software device in classifying a CXR as normal or abnormal. A manufacturer-defined pre-set threshold of 0.5 was applied to the probability scores to classify a CXR as normal or abnormal by the AI. The definitions of true positive (TP), false positive (FP), true negative (TN), and false negative (FN) cases at the CXR-level in the context of the statistical analysis were:

1. **TP**: Both AI and ground truth classified a CXR as abnormal.
2. **TN**: Both AI and ground truth classified a CXR as normal.
3. **FP**: AI classified a CXR as abnormal, but the ground truth was normal.
4. **FN**: AI classified a CXR as normal, but the ground truth was abnormal.

The main outcome measure of interest was the negative predictive value (NPV). Sensitivity, specificity, positive predictive value (PPV), and area under the receiver operating characteristics curve (AUROC) are also reported. Point estimates and 95% confidence intervals (CIs) based on the exact binomial method are reported for PPV, NPV, sensitivity, and specificity.^22^ AUROC was estimated based on empirical method, and DeLong’s 95% CI of AUROC is reported.^23^^,^^24^ Since NPV is dependent on the prevalence of the disease and not just on the accuracy of the device, a sensitivity analysis of NPV by varying assumptions of the prevalence of normal CXR images is also reported.^25^ A failure analysis of false negative results was also performed. Any identifiable patient information was removed, and anonymized patient identifiers were allocated to each patient before extracting the variables for statistical analysis into a Microsoft Excel file. All statistical analyses were done using R version 4.2.1.^26^

### Ethical considerations

This service evaluation was conducted using a set of retrospectively collected CXR images. As such, there was no requirement for ethics committee approval or informed consent. Frimley Health Internal AI Working Group approved this service evaluation.

## Results

**Figure 1.**
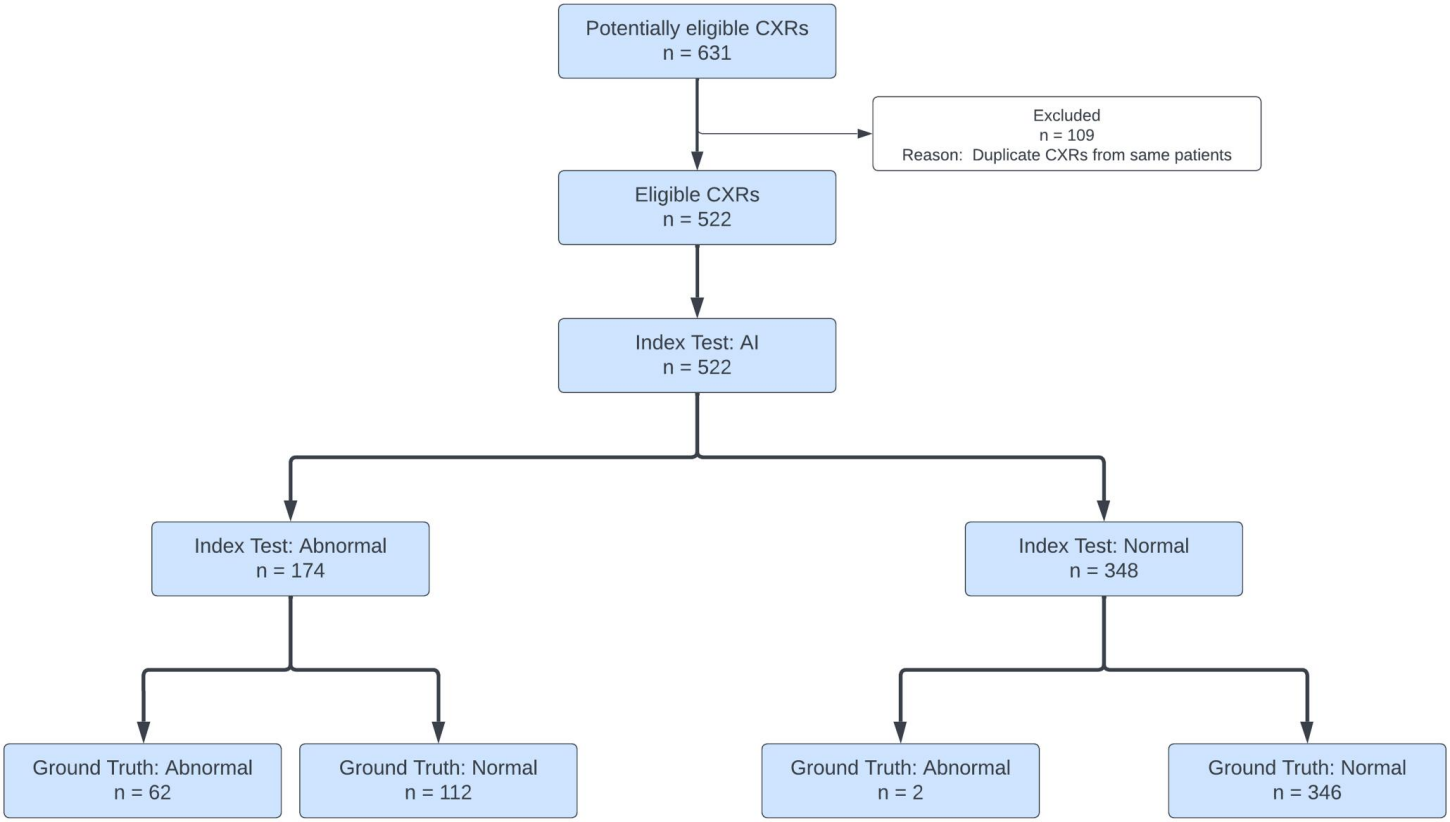
Data flow diagram.

A total of 631 CXR images were randomly selected for this service evaluation. One hundred nine duplicate CXR images were excluded. Thus, a total of 522 CXR images from 522 patients were considered for this service evaluation. A diagram reporting the flow of CXR images selected for this service evaluation is shown in Figure 1. The mean and median age of the patients were 62 (standard deviation: 18) and 64 (inter-quartile range: 49-77) years, respectively. A total of 305 (58.43%) patients were female. Of the 522 patients, ethnicity information was available for 472; 437 (92.58%) were White, 17 (3.6%) were Asian, 12 (2.54%) belonged to “other” or “mixed” category of ethnicity, and 6 (1.28%) were Chinese.

**Figure 2.**
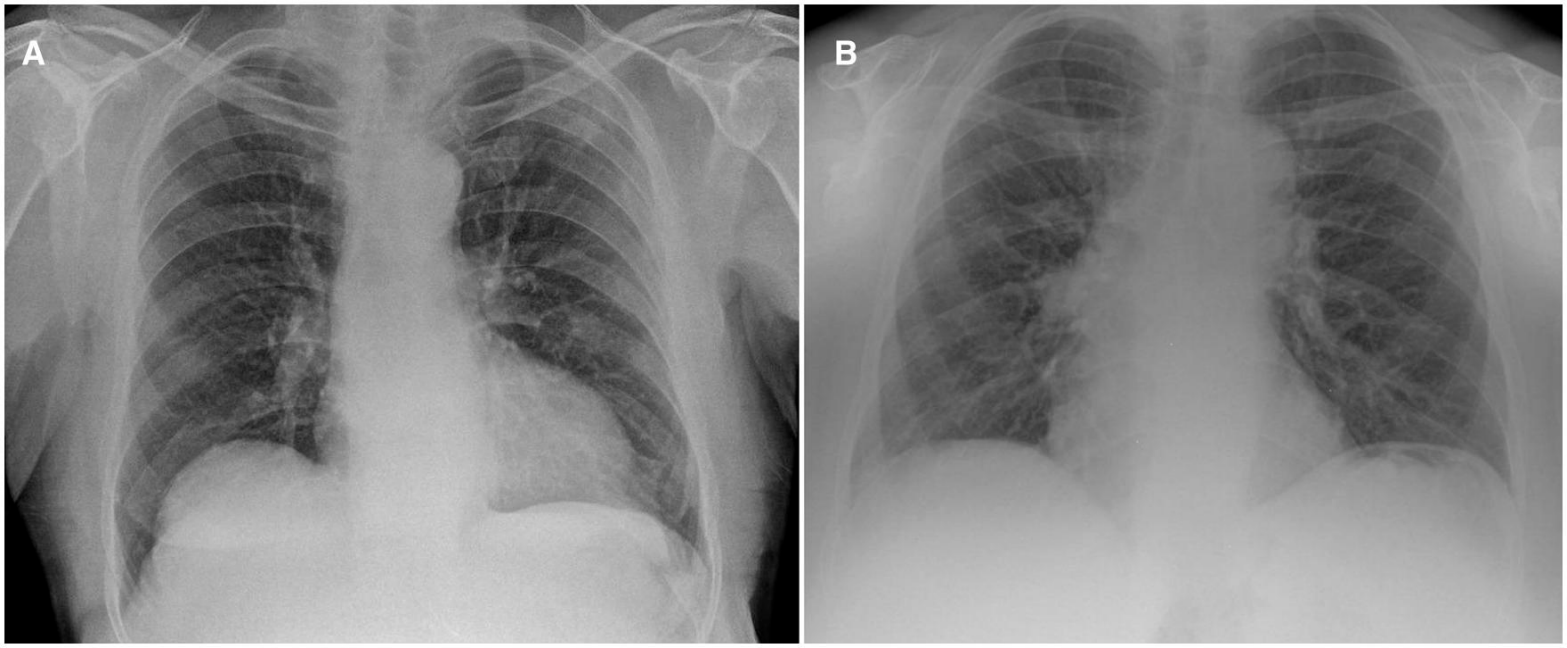
(A) Subtle shadowing in left upper chest, which later turned out to be a case of malignancy. (B) Right hilar fullness, but subsequent CT chest was normal.

There were no indeterminate results or missing values in AI results. As per ground truth, 458 (87.74%) of the 522 CXR images were normal, and the rest, 64 (12.26%), were abnormal. qXR classified 348 (66.67%) of the CXR images as normal and the rest 174 (33.33%) as abnormal. The Cohen’s kappa was found to be 0.94 (95% CI, 0.89-0.98) and the Gwet’s AC1 was found to be 0.98 (95% CI, 0.97-1.00) between original radiology report and the ground truth (consensus of 2 radiologists), indicating a very good agreement for presence or absence of remarkable abnormalities in the CXRs. The two CXRs in which the false negative results were observed are shown in Figure 2. The contingency table of the classification results is shown in Table 1.

**Table 1.** Contingency table showing classification results.

	Ground truth: abnormal	Ground truth: normal	Total
**AI: abnormal**	62	112	174
**AI: normal**	2	346	348
**Total**	64	458	522

Abbreviation: AI = artificial intelligence.

**Figure 3.**
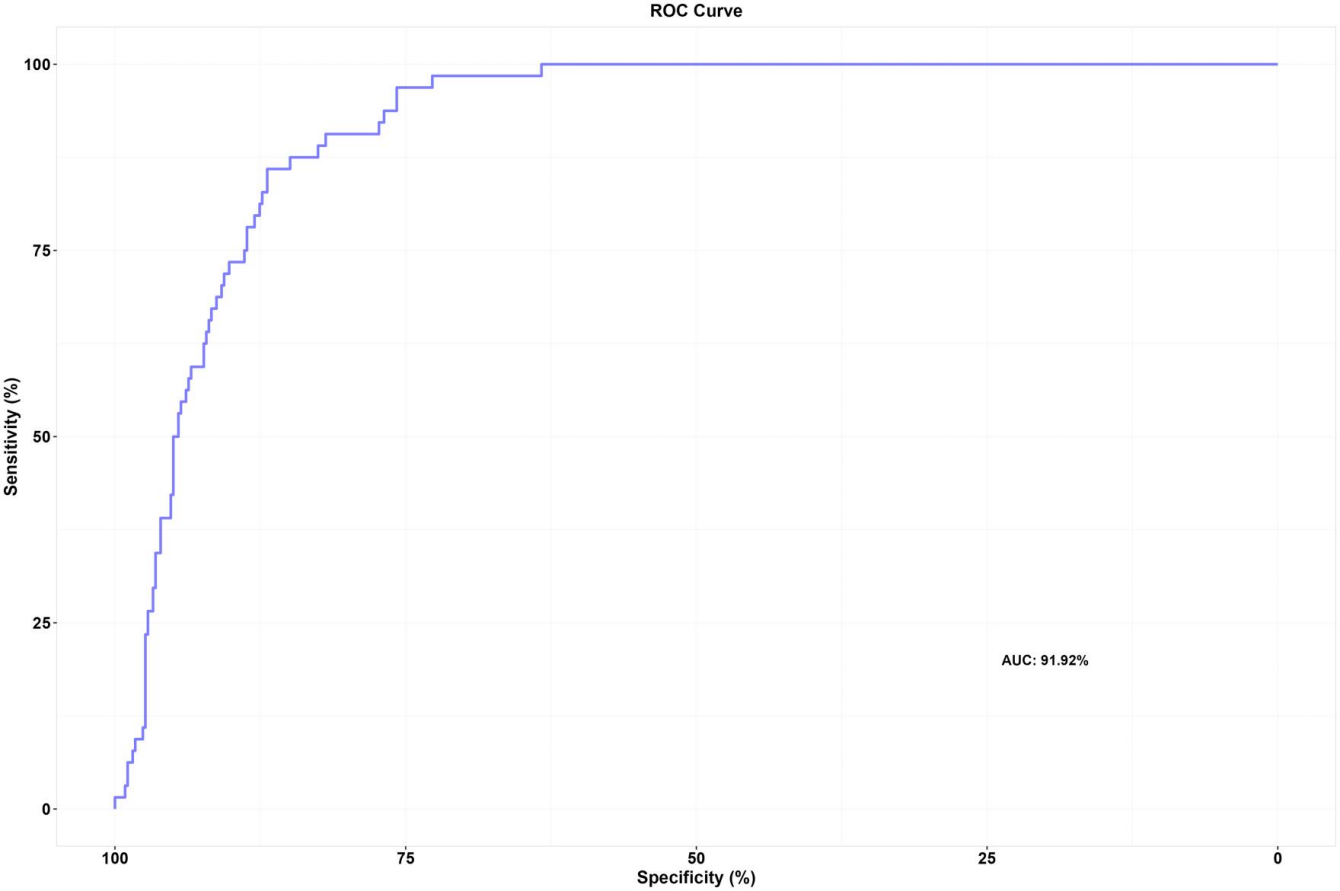
ROC curve of the AI software device in classifying a CXR as abnormal or normal. AI = artificial intelligence; CXR = chest X-ray; ROC = receiver operating characteristics.

NPV was estimated to be 99.43% (95% CI, 97.94-99.93). The sensitivity, specificity, and PPV of the AI software device were estimated to be 96.88% (95% CI, 89.16-99.62), 75.55% (95% CI, 71.34-79.42), and 35.63% (95% CI, 28.53-43.23), respectively. The empirical AUROC point estimate was 91.92% (95% CI, 89.38-94.45), and the receiver operating characteristics (ROC) curve is shown in Figure 3.

### Sensitivity analysis of NPV

**Figure 4.**
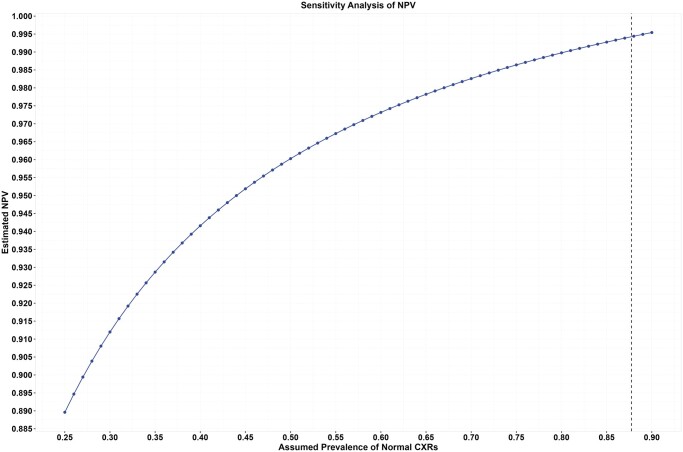
Sensitivity analysis of NPV at varying assumptions of prevalence of normal CXRs. Dotted line indicates the observed prevalence in the sample. CXR = chest X-ray; NPV = negative predictive value.

The prevalence of normal CXR images in the sample was about 87.74% (458/522). NPV and PPV are dependent on the prevalence of the target condition. A sensitivity analysis was conducted by assuming a range of the prevalence of normal CXR images from 25% to 90% and by assuming the sensitivity and specificity of the device to be the estimates reported earlier in this article. The NPV estimates ranged from 88.96% to 99.54% as the prevalence of normal CXR images increased from 25% to 90% (Figure 4).

### Failure analysis

The two false negative cases were again reviewed after the analysis was completed, and it was found that one of them had a follow-up CT, which indicated no abnormalities, and another one was a missed lung nodule, which turned out to be cancerous in subsequent investigations (Figure 2). We processed the missed lung nodule CXR image with a newer version (qXR version 4.0) of the AI device, and the “missed” nodule in CXR was detected correctly by the more recent version. This indicates that AI software devices may have different versions and performance may differ between different versions of the same device.

## Discussion

The AI software device was able to correctly identify 62 of the 64 (sensitivity of 96.38%) CXR images containing radiological abnormalities and 346 out of the 458 (specificity of 75.55%) normal CXR images. The relatively low specificity is likely because the AI is trying to find as many identifiable abnormalities in a CXR, which may include abnormalities not requiring any special attention or mention from the radiologists, such as small and long-standing nodules, old rib fractures, etc. Some of the occurrences of false positives were due to the presence of nipple shadows, which the AI identified as opacity, thus classifying them as “abnormal.” It is possible that some of the false positive results occurred because AI flagged the CXRs as abnormal, but the radiologists deemed that these CXRs are essentially normal because they did not warrant a follow-up investigation. A study conducted in Denmark using a similar AI software device using CXR images sourced from patients reporting to emergency departments, inpatients, and OPs (prevalence of normal CXR images in this study was about 28%) reported a sensitivity of approximately 99.1% and specificity of 28.0%.^8^ There are also other studies reporting the accuracy of AI in the use-case of normal versus abnormal CXR stratification^8–13^^,^^27^ or in identifying suspicious abnormalities.^28^ Our work adds further evidence to the usefulness of such AI algorithms although it has to be noted that there is probably no standard definition of what is a normal CXR because researchers use different definitions and this can make the comparisons between different research works difficult.

From the context of a potential autonomous reporting of “normal” CXR images, false negatives may be relatively more harmful errors than false positives, assuming that false positive results may still be reviewed by qualified radiologists in such a hypothetical workflow where normal CXRs detected by AI are deprioritized for reporting or redirected to an alternative non-radiologist workflow. Hence, a high NPV is desirable for AI software devices that are intended for identifying and triaging normal CXRs. A high NPV indicates that radiologists or other intended users can be more confident when the AI flags a CXR as normal. In our sample sourced from GP and OP sources with a relatively high prevalence of normal CXR images, we found that the AI software device yielded a high NPV of 99.43% (95% CI, 97.94-99.93). A recently published study of the same device conducted in the UK reported an NPV of about 99.2% (95% CI, 97.2-99.7).^12^ Another study conducted in a radiological diagnostic centre in India reported an NPV of about 98.9% (95% CI, 98.1-99.0).^27^ The UK study utilized a sample of GP and OP referral CXRs, whereas the study from India was based on OP or health check-up CXRs. Our results are comparable to the reported estimates of both studies although it is to be noted that study populations may be different and statistical comparison with a meta-analysis is out-of-scope for this evaluation. False positive results can also have serious consequences, and users of AI tools, such as radiologists, should be aware of the possibility of false positives to enable optimal use of these decision support tools. Overtesting and overdiagnosis may end up as consequences of false positive results. If the AI is intended to be used only for deprioritizing or removing AI-flagged normal CXRs from the radiologists’ workflow (hypothetical scenario) and that the AI-flagged abnormal CXRs are reported as per routine care (radiologists being blinded to AI results), then it can be assumed that AI-induced false positive outcomes for the patients can be mitigated at least partly.

A careful adjustment of the threshold for classifying a CXR as abnormal will help in reducing false negatives. For this service evaluation, a default threshold of 0.5 was applied to the probability score to classify a CXR as abnormal or normal. If the threshold were set at 0.15, then there would have been no false negatives, but at the cost of decreased specificity of 51.97% (95% CI, 47.28-56.62) and thus increased number of false positives. This is suggestive that threshold adjustments may be needed to achieve optimal performance of the AI device before deploying such devices for routine clinical use.^29^ In our failure analysis, we also noticed that a different version of AI algorithm can behave differently from other versions. Typically, newer versions are more accurate than older versions.^30^

CXR images from inpatient and emergency departments may have a lower prevalence of normal CXR images as compared to those sourced from GP and OP pathways. In settings where the prevalence of normal CXR images can be safely assumed to be relatively on the higher side, the sensitivity analysis suggested that the AI device may achieve high NPVs of more than 95%.

In a previous study of the same device, it was reported that the device may facilitate faster reporting of CXR examinations in real-time if used as an assistance device for radiologists.^27^ The removal of even a tiny proportion of all CXR images has the potential to reduce the workload of radiologists, thereby allowing them to dedicate time to more serious examinations.^16^ If, hypothetically, we assume that AI will flag about 50% of CXRs as normal, and these AI-flagged normal CXRs are then transferred to a radiographer-based reporting workflow, the radiologists may be able to save 2 h in a typical 4-h reporting session.^31^ There could also be negative impacts on radiologist efficiency such as alarm fatigue.^32^^,^^33^

There are several limitations for our service evaluation. A larger sample consisting of consecutive CXR examinations would have been more appropriate for a comprehensive evaluation. Ours was a pilot service evaluation and used a smaller sample due to logistical reasons. We acknowledge that there is a risk that the sample may not be representative of the true spectrum and true prevalence of the disease. We conducted a sensitivity analysis by varying the assumptions of the prevalence of normal CXR images from 25% to 90%, and we found that the NPV of the device may range from 88.96% to 99.54%, assuming the same sensitivity and specificity we estimated from the sample. The latter assumption is also not free from limitations, especially given the relatively small sample size (*n* = 64) of actual abnormal CXR images. The radiologists who established the ground truth are from a single centre and this may not be ideal for a robust ground truth establishment. We did not perform subgroup analysis based on age and gender. A similar study of the same AI software device conducted in another NHS Trust reported comparable NPV as indicated by reasonable overlap of the 95% CIs when stratified by gender or age groups.^12^ Our purpose was to evaluate the performance of the AI in triaging normal from abnormal CXRs and thus our major focus was in NPV estimation and failure analysis of false negative results. For this reason, an abnormality-level analysis was out of scope for this evaluation.

## Conclusion

In this service evaluation, it was found that the AI software device was able to classify CXR images into normal and abnormal with a high NPV of approximately 99.43%. Such a tool with high NPV has the potential to reduce radiologist workload. Further research studies using a prospective sample of consecutive CXR images with sufficient statistical power may be needed to generate more evidence on the safety and effectiveness of such devices and operational impact on radiologist workload and CXR reporting turnaround times.

## Data Availability

The CXR images used for this service evaluation are confidential and cannot thus be shared. The de-identified structured dataset used for statistical analysis may be shared upon reasonable request.

## References

[1] Speets AM , van der Graaf Y, Hoes AW, et al Chest radiography in general practice: indications, diagnostic yield and consequences for patient management. Br J Gen Pract J R Coll Gen Pract. 2006;56(529):574-578. PMC1874520 16882374

[2] Stevens BJ , Skermer L, Davies J. Radiographers reporting chest X-ray images: identifying the service enablers and challenges in England, UK. Radiogr (London, Engl 1995). 2021;27(4):1006-1013. 10.1016/j.radi.2021.03.006 33875356

[3] NHS England. Diagnostic imaging dataset—statistical report [Internet]. Version 1. 2023. Accessed May 18, 2023. https://www.england.nhs.uk/statistics/wp-content/uploads/sites/2/2023/05/Statistical-Release-18th-May-2023-PDF-471KB-1.pdf

[4] Commission CQ. A national review of radiology reporting within the NHS in England [Internet]. Cited September 05, 2023. https://www.cqc.org.uk/sites/default/files/20180718-radiology-reporting-review-report-final-for-web.pdf

[5] Harolds JA , Parikh JR, Bluth EI, Dutton SC, Recht MP. Burnout of radiologists: frequency, risk factors, and remedies: a report of the ACR commission on human resources. J Am Coll Radiol. 2016;13(4):411-416. 26768546 10.1016/j.jacr.2015.11.003

[6] Al Rekabi A , Chen M, Patel N, et al Well-being and burnout amongst interventional radiologists in the United Kingdom. Cardiovasc Intervent Radiol. 2023;46(8):1053-1063. 37380792 10.1007/s00270-023-03455-5 PMC10382397

[7] Krupinski EA. Current perspectives in medical image perception. Atten Percept Psychophys. 2010;72(5):1205-1217. 10.3758/APP.72.5.1205 20601701 PMC3881280

[8] Plesner LL , Müller FC, Nybing JD, et al Autonomous chest radiograph reporting using AI: estimation of clinical impact. Radiology. 2023;307(3):e222268. 10.1148/radiol.222268 36880947

[9] Yoo H , Kim EY, Kim H, et al Artificial intelligence-based identification of normal chest radiographs: a simulation study in a multicenter health screening cohort. Korean J Radiol. 2022;23(10):1009-1018. 36175002 10.3348/kjr.2022.0189 PMC9523233

[10] Hwang EJ , Park S, Jin K-N, et al; DLAD Development and Evaluation Group. Development and validation of a deep learning-based automated detection algorithm for major thoracic diseases on chest radiographs. JAMA Netw Open. 2019;2(3):e191095. 30901052 10.1001/jamanetworkopen.2019.1095 PMC6583308

[11] Vogado L , Araújo F, Neto PS, Almeida J, Tavares JMRS, Veras R. A ensemble methodology for automatic classification of chest X-rays using deep learning. Comput Biol Med. 2022;145:105442. https://www.sciencedirect.com/science/article/abs/pii/S0010482522002347?via%3Dihub 35344867 10.1016/j.compbiomed.2022.105442

[12] Blake SR , Das N, Tadepalli M, et al Using artificial intelligence to stratify normal versus abnormal chest X-rays: external validation of a deep learning algorithm at East Kent Hospitals University NHS Foundation Trust. Diagnostics. 2023;13(22):3408. 37998543 10.3390/diagnostics13223408 PMC10670411

[13] Mansoor A, , Schmuecking I, , Ghesu FC, et al Large-Scale Study on AI's Impact on Identifying Chest Radiographs with No Actionable Disease in Outpatient Imaging. Acad Radiol. 2024. 10.1016/j.acra.2024.06.031 38997881

[14] Recht M , Bryan RN. Artificial intelligence: threat or boon to radiologists? J Am Coll Radiol. 2017;14(11):1476-1480. 28826960 10.1016/j.jacr.2017.07.007

[15] van Leeuwen KG , de Rooij M, Schalekamp S, van Ginneken B, Rutten MJCM. How does artificial intelligence in radiology improve efficiency and health outcomes? Pediatr Radiol. 2022;52(11):2087-2093. 34117522 10.1007/s00247-021-05114-8 PMC9537124

[16] Dyer T , Dillard L, Harrison M, et al Diagnosis of normal chest radiographs using an autonomous deep-learning algorithm. Clin Radiol. 2021;76(6):473.e9-473.e15. 10.1016/j.crad.2021.01.015 33637309

[17] Annarumma M , Withey SJ, Bakewell RJ, Pesce E, Goh V, Montana G. Automated triaging of adult chest radiographs with deep artificial neural networks. Radiology. 2019;291(1):196-202. 10.1148/radiol.2018180921 30667333 PMC6438359

[18] Tang Y-X , Tang Y-B, Peng Y, et al Automated abnormality classification of chest radiographs using deep convolutional neural networks. NPJ Digit Med. 2020;3(1):70. 10.1038/s41746-020-0273-z 32435698 PMC7224391

[19] NICE. Recommendations | Artificial intelligence-derived software to analyse chest X-rays for suspected lung cancer in primary care referrals: early value assessment [Internet]. Cited November 24, 2023. https://www.nice.org.uk/guidance/hte12/chapter/1-Recommendations

[20] Putha P , Tadepalli M, Reddy B, et al Can artificial intelligence reliably report chest X-rays?: radiologist validation of an algorithm trained on 1.2 million X-rays. CoRR [Internet]. abs/1807.0. arXiv, http://arxiv.org/abs/1807.07455, 2018, preprint: not peer reviewed.

[21] Frimley Health NHS Foundation Trust. [Internet]. Cited September 25, 2023. https://www.fhft.nhs.uk/

[22] Clopper CJ , Pearson ES. The use of confidence or fiducial limits illustrated in the case of the binomial. Biometrika. 1934;26(4):404-413.

[23] Delong ER , Delong DM, Clarke-Pearson DL. Comparing the areas under two or more correlated receiver operating characteristic curves: a nonparametric approach. Biometrics. 1988;44(3):837-845. 3203132

[24] Zhou XH , Obuchowski NA, McClish DK. Statistical Methods in Diagnostic Medicine. Wiley. 2011:1-545.

[25] Parikh R , Mathai A, Parikh S, Chandra Sekhar G, Thomas R. Understanding and using sensitivity, specificity and predictive values. Indian J Ophthalmol. 2008;56(1):45-50. 18158403 10.4103/0301-4738.37595 PMC2636062

[26] R: The R Project for Statistical Computing. [Internet]. Cited September 25, 2023. https://www.r-project.org/

[27] Govindarajan A , Govindarajan A, Tanamala S, et al Role of an automated deep learning algorithm for reliable screening of abnormality in chest radiographs: a prospective multicenter quality improvement study. Diagnostics. 2022;12(11):2724. 36359565 10.3390/diagnostics12112724 PMC9689183

[28] Maiter A , Hocking K, Matthews S, et al Evaluating the performance of artificial intelligence software for lung nodule detection on chest radiographs in a retrospective real-world UK population. BMJ Open. 2023;13(11):e077348. 10.1136/bmjopen-2023-077348 PMC10632826 37940155

[29] WHO. Calibrating CAD for TB [Internet]. 2021. Cited May 15, 2024. https://tdr.who.int/activities/calibrating-computer-aided-detection-for-tb

[30] Qin ZZ , Barrett R, Ahmed S, et al Comparing different versions of computer-aided detection products when reading chest X-rays for tuberculosis. PLOS Digit Health. 2022;1(6):e0000067. 36812562 10.1371/journal.pdig.0000067 PMC9931298

[31] Radiologists TRC of. RCR [Internet]. 2022. Cited November 28, 2023. https://www.rcr.ac.uk/media/lmojozhq/rcr-publications_radiology-reporting-figures-for-service-planning-2022_november-2022.pdf

[32] Ancker JS , Edwards A, Nosal S, Hauser D, Mauer E, Kaushal R, with the HITEC Investigators [Internet]. Effects of workload, work complexity, and repeated alerts on alert fatigue in a clinical decision support system. BMC Med Inform Decis Mak. 2017;17(1):36. 10.1186/s12911-017-0430-8 28395667 PMC5387195

[33] Komorowski M , Celi LA. Will Artificial Intelligence Contribute to Overuse in Healthcare?. Crit Care Med. 2017;45(5):912-913. 28410309 10.1097/CCM.0000000000002351 PMC5679196

